# A Singular Case of Complete Closure of the Vagina, with Subsequent Conception, and Safe Delivery at the Full Period of Utero-gestation

**Published:** 1847-02

**Authors:** Robert P. Simmons

**Affiliations:** Saint Louis, Mo.


MEDICAL EXAMINER

AND

RECORD OF MEDICAL SCIENCE.

NEW SERIES.—No. XXVI.—FEBRUARY, 1847.

---

### ORIGINAL COMMUNICATIONS.

*A singular case of complete closure of the Vagina, with subsequent conception, and safe delivery at the full period of utero-gestation.* By ROBERT P. SIMMONS, M. D., St. Louis, Mo.

Some time towards the end of March, 1846, I was consulted by Mrs. E. B. W., a respectable married woman of this city, in reference to a mal-condition of the sexual organs, which she said rendered her unhappy in her domestic relations, and which she greatly desired to have remedied, should it be thought practicable. It not being convenient at the time to learn the history, or to enter upon an examination of the case, I promised to wait on her in a few days. Accordingly, on the second day of the following month, (April,) accompanied by my friend Dr. John Barnes, whom I invited to be present, I visited Mrs. W. for the purpose of making an examination of her condition. Before pro ceeding to do so, she detailed to us in substance the following history:

In the month of July, 1844,she was confined with her first and only child; had a laborious and very protracted labour, in which she suffered almost beyond endurance; the child was still-born and unusually large; at the time of its birth she was exhausted and unconscious of what had happened. In a very few days after her accouchement, acute phlegmonous inflammation invaded the soft parts, terminating in sloughing, with excessive and longcontinued suppuration; and it was not till the middle of October following, that she could begin to date her recovery. Some time during the succeeding month (November) she became aware for the first time of her peculiar and unhappy condition—a complete closure of the vagina, which she observed "has continued unchanged to the present time."

Mrs. W. was attended in her accouchement by a medical gentleman of this city, who corroborates all she has said touching the severity of her labour, but, for some cause or other, he lost sight of the patient shortly afterwards, and can give no account of her subsequent and protracted illness.

Mrs. W. is a native of Ireland, aged 40 years, sanguineo-nervous temperament, possesses a good constitution, and never, as she informed us, failed to menstruate at her regular monthly periods, as well since as previous to her long confinement in 1844, except when she was pregnant, and at the period immediately preceding this examination, which she missed, and which ought to have been, as she said, about the middle of March, 1846.

Mrs. W. willingly submitted to an examination, with the hope that something could be done to remedy the defect. Accordingly she was placed on the bed, with the thighs elevated and the parts properly adjusted to the light. On proceeding to the examination, we could perceive nothing defective or unnatural in the labia externa, clitoris and fourchette, but on separating the labia to their fullest extent, we found the nymphæ almost entirely obliterated, and a dense fibrous structure presenting to our view, extending from the orifice of the urethra to the fourchette, and across from the base of one labium to that of the other, blocking up the mouth of the vagina completely, and resembling more than anything in appearance the palm of one's hand, when in an extended position. Dim tortuous lines of cicatrices were perceptible, and the whole surface of this structure presented to the touch a smooth, dense, elastic feel, but no opening or cavity whatever could be perceived. Impressed as we were, however, that an orifice or orifices did exist, from the circumstance of the menstrual discharge not being interrupted, we continued the examination for more than an hour with the greatest care and minuteness; used the smallest sized probes, applied them to every point of this structure and in every direction to its surface; also to the rugæ of mucous tissue lining the clitoris, fourchette and orifice of urethra, but we were unable to discover a single orifice or fissure communicating with the cavity of the vagina or uterus. The orifice of the urethra was of usual size, and occupied its natural situation—admitting the easy introduction of a medium sized catheter and its ready passage to the bladder. Our attention was then directed to the exploration of the urethral canal, believing it possible that a fistulous orifice might be found near the opening of that tube, through which the menstrual fluid made its way from the uterus. Probes of various forms and sizes were used, but without obtaining the object of our pursuit; and ascertaining from the woman, that at no time during her menstrual flow did she perceive the discharges, either per urethram or per anum, even in the slightest degree tinctured with that fluid, we declined the further prosecution of our inquiry.

Unwilling at that time, to give a conclusive opinion with regard to the propriety of an operation for the restoration of the vaginal tube, I deferred doing so from time to time, until the latter part of July following, when the husband of this woman called to inform me that his wife began to suspect herself pregnant. I supposed at once that her fears arose from the suppression of menstruation, which I was aware had not appeared since the middle of February, this period being about six weeks previous to my first examination. I advised the man to go home and say to his wife that she was mistaken, assuring him at the same time that such an event was a total impossibility; and he, poor man, having too long had sufficient proofs of his wife's inaptitudes in this way, readily acquiesced with me in opinion. I called to see Mrs. W. on the 8th of August following; perceived that she had increased in size since I saw her last, and was about as large as one at the middle period of gestation. She repeated to me that she had not menstruated since the middle of February, and said that "she felt something move within her belly." The first time, as near as she could recollect, was about the middle of July.

Previously immersing my hand in cold water, I applied it to the abdomen, causing her suddenly to change her position from side to side, but I could perceive no motion. I then questioned her more critically than I had done, with regard to her sensations and aptitudes on the embrace of her husband. She declared to me her total incompetency to afford her husband any reciprocity in sexual intercourse, and also assured me that at no time since her misfortune in 1844, did she ever experience any other sensation than that of disrelish on his embrace. She further stated that she had a kind and effectionate husband, and it was on account of his privation alone, that she was induced to make her condition known to me. "If I am in the family way, it is as much a mystery to me as it is to you, Doctor, but from the way I feel, there *surely must be something living within me.*"

The case now became exceedingly interesting, and although I still doubted the possibility of pregnancy, I offered no further objections to her own diagnosis, based as it was on evidence so conclusive to her, and which, the issue of the case has proven, was more certain than the lights by which I was governed in physiological science.

Mrs. W. requested me to keep her case in view, and if she were pregnant to attend her in her confinement; assuring me at the same time that she would look to no other source, except to her God, for help in her approaching tribulation! I continued to visit her occasionally, and as often found her increasing in size, and the abdomen presenting the feel and contour of one containing a growing fœtus in utero.

Fully satisfied as I was with regard to the actual condition of this woman, as herein stated, yet I felt solicitous for additional testimony, should she turn out to be pregnant, and with that view I obtained the consent of Mrs. W. to allow another professional friend, Dr. Cornelius Campbell, to make an examination of her case. He accordingly did so, with me, on the 3d day of September last. On this occasion we made the same close and minute examination that was done by Dr. Barnes and myself on the 2d of April,—used the smallest sized probes of every shape and form,—applied them in every direction and to every point of the fibrous structure blocking up the vagina; also between the folds of mucous tissue lining the labia pudendi, &c. &c., but we were equally unsuccessful as I had been before, for no passage or orifice whatever could be found communicating with the vagina.

The catheter was readily introduced into the bladder, as on a former occasion, but we could find no track from the urethra to the vagina or to any other cavity. Neither was there any malformation of the anus or fistulous orifices about its margin. This was the last ocular examination I had of the case; and I must here express my extreme regret that I was not permitted to have a cast or drawing taken of the parts, by which a clearer and more accurate idea could have been conveyed of the appearance and condition I have endeavoured to describe.

On the 20th of October last I was requested to visit Mrs. W. as a patient. She had been very ill the night previous, and complained of distressing pains in the back and loins, rendering her unable to be easy in any position more than fifteen minutes at a time. She was as large as one very near the full period of gestation, and by placing my hand on the abdomen I felt, for the first time, the motion of the fœtus. During the continuance of a pain I made an examination per os externum, and found the structure referred to presenting to the touch the same feel that it did on former examinations. There was no fulness or distension of the parts which the descent of a fœtus and collected waters within the membrane would indicate; I was, however, fully convinced that the poor woman was pregnant, and presuming, on her own calculations, that she had not arrived at her full period of gestation, I took from the arm ℥xx. of blood, and gave Tinct. acetatis opii gutt. xxx. She was much relieved, and continued comparatively easy for several days, but the annoying pains returned, and continued with more or less severity up to the time of her accouchement.

On the 19th of November, at 6 o'clock, A. M., I was summoned to visit Mrs. W. in haste. On entering her apartment I found her in strong labour, and on making an examination per os externum, I discovered a fulness with great tension and pressing down of the smooth fibrous structure blocking up the vagina; and this condition of the parts increased on the return of every succeeding pain, till in the space of two hours I could perceive evident fluctuation of the waters within the membranes, and by forcible pressure with the fingers could feel a hard body low down in the pelvis, which I supposed was the head of the child.

Not feeling disposed to assume the entire responsibility of this extraordinary case, and besides desiring to afford the professional gentlemen who had previously examined the condition of this woman, Drs. Barnes and Campbell, an opportunity of witnessing the issue of the case, they were ordered to be sent for. In the mean time the parturient pains continuing with powerful force, the patient was placed on her back, with the thighs separated, and opening the labia-externa with the thumb and fingers of the left hand, I carefully divided the integuments with the scalpel to the extent of one inch, commencing the incision at the most prominent part of the tumour, and on a direct line from the orifice of the urethra to the perineum. The structure divided was about three-fourths of an inch in thickness, and of a firm fibrous texture, clearly indicating that the occlusion was not the result of simple adhesion of the sides of the vagina, but the new formation of tendinous tissue. Whether the membranes containing the liquor amnii had previously ruptured, or whether they were punctured with the point of the scalpel, it is impossible for me to know; at any rate, on passing through the structure with the knife, there was a free jet and flow of water for a moment, and also considerable hemorrhage, but this was not to an alarming extent, having its source principally in the great vascularity of the parts surrounding and closing the vagina.

I was now able to ascertain the condition of the os uteri—found it dilated to the size of a twenty-five cent piece, the margin thin and of a natural feel, with the head of the child presenting. By this time Dr. Campbell arrived, (Dr. Barnes not having received the notice,) when we dilated the incision previously made to the extent of two inches, by carefully dividing fibre by fibre, with a well guarded probe-pointed bistoury, cutting both anteriorly and posteriorly. The pains continued with wonderful force, and the os uteri dilating, we soon were able to perceive the hairy scalp of the child, and ascertain more clearly its position, which was the posterior fontanelle to the symphysis pubis. The soft parts readily and safely dilated as the head advanced; and although the labour was both tedious and laborious, until the head emerged from the inferior strait of the pelvis, not an untoward event occurred, and the woman was safely delivered at five o'clock, P. M. same day, of a well formed and living female child, weighing 8 pounds. At this date (January 2d, 1847) both mother and child are living and doing well, and the poor woman feels doubly compensated for all her past afflictions, by the entire removal of her physical impotency, and the new source of moral happiness which has been lighted up on becoming a mother.

It may be proper to add, that great care was paid to the subsequent management of this case, and by means of a tent and other necessary applications, the newly formed vagina has been maintained. Suppuration has entirely ceased, and new mucous tissue partially formed on the internal sides of the divided structure.

The physiological questions involved in this case are—how did the menstrual fluid make its exit from the uterus, and by what law of the animal economy did conception take place? The woman's own statement, (her husband also concurring in the same facts) was, that since recovery from her long confinement in 1844, which was about the end of November of that year, her condition had been at no time different from what it was at my first and subsequent examinations; yet, notwithstanding, she menstruated regularly up to the middle of February, and at that time was as much unwell as usual.

She could give no account of it, except that the discharge issued from between the labia externa was small in quantity, continued about three days, and during these periods she never experienced any pain or difficulty whatever. Here I must express another regret—that in the investigation of this case, I never had an opportunity of observing the appearance of the parts during menstruation, which at the time I made my first examination it was my settled purpose to do at the next or some period of their flow, not having the most distant idea that pregnancy was the cause of their supppression.

Was there a vicarious secretion from the inner surface of the labia pudendi and contiguous parts, or did the menstrual fluid permeate through the fibrous structure blocking up the vagina?

I am inclined to adopt the latter opinion, although we were unable, on the minutest examination, to detect that condition of the surface which such a process would indicate.

In contemplating the curious phenomenon of conception in this case, I find on the very outset of the inquiry, the physiological views I had long entertained on the subject are subverted, and not feeling prepared to attempt an elucidation of that wonderful and mysterious function of organic life—conception—on any other principle than that of the actual presence of the semen masculinum within the cavity of the uterus, I will submit the rationale of this singular case to those who are deeper read in nature's laws, and whose province it is to demonstrate the occult mysteries in physiological science.

*Saint Louis, Mo.*, January 2d, 1847.

